# Histone Methylation Regulation in Neurodegenerative Disorders

**DOI:** 10.3390/ijms22094654

**Published:** 2021-04-28

**Authors:** Balapal S. Basavarajappa, Shivakumar Subbanna

**Affiliations:** 1 Division of Analytical Psychopharmacology, Nathan Kline Institute for Psychiatric Research, Orangeburg, NY 10962, USA; subbanna.shivakumar@nki.rfmh.org; 2 New York State Psychiatric Institute, New York, NY 10032, USA; 3 Department of Psychiatry, College of Physicians & Surgeons, Columbia University, New York, NY 10032, USA; 4 New York University Langone Medical Center, Department of Psychiatry, New York, NY 10016, USA

**Keywords:** epigenetics, Alzheimer’s disease, Parkinson’s disease, Huntington’s disease, Amyotrophic lateral sclerosis, neuronal loss and alcohol

## Abstract

Advances achieved with molecular biology and genomics technologies have permitted investigators to discover epigenetic mechanisms, such as DNA methylation and histone posttranslational modifications, which are critical for gene expression in almost all tissues and in brain health and disease. These advances have influenced much interest in understanding the dysregulation of epigenetic mechanisms in neurodegenerative disorders. Although these disorders diverge in their fundamental causes and pathophysiology, several involve the dysregulation of histone methylation-mediated gene expression. Interestingly, epigenetic remodeling via histone methylation in specific brain regions has been suggested to play a critical function in the neurobiology of psychiatric disorders, including that related to neurodegenerative diseases. Prominently, epigenetic dysregulation currently brings considerable interest as an essential player in neurodegenerative disorders, such as Alzheimer’s disease (AD), Parkinson’s disease (PD), Huntington’s disease (HD), Amyotrophic lateral sclerosis (ALS) and drugs of abuse, including alcohol abuse disorder, where it may facilitate connections between genetic and environmental risk factors or directly influence disease-specific pathological factors. We have discussed the current state of histone methylation, therapeutic strategies, and future perspectives for these disorders. While not somatically heritable, the enzymes responsible for histone methylation regulation, such as histone methyltransferases and demethylases in neurons, are dynamic and reversible. They have become promising potential therapeutic targets to treat or prevent several neurodegenerative disorders. These findings, along with clinical data, may provide links between molecular-level changes and behavioral differences and provide novel avenues through which the epigenome may be targeted early on in people at risk for neurodegenerative disorders.

## 1. Introduction

Neurodegenerative (ND) disorders are among the leading bases of disability and death worldwide [1,2,3]. The neurodegeneration process involves progressive atrophy of neurons, leading to loss of neuronal connectivity and function followed by its demise, thereby adversely affecting brain function. Despite decades of basic and clinical research, most strategies designed to reverse degenerative brain diseases are analgesic. This is not surprising as neurodegeneration progresses quietly for decades before the appearance of symptoms. Most important advances in sequencing technologies have allowed the mapping of transcriptomic patterns in human postmortem brain tissues in various ND disorders, including in vitro and in vivo cell and animal models. These investigations facilitated the discovery of classical neurodegeneration pathways and uncovered novel targets, including synaptic degeneration in the majority of ND, that share several puzzling characteristics. For example, although large patient populations’ intense genetic evaluation has been performed, a substantial proportion of ND incidents have no known genetic origin [4], and only a few neuro-pathophysiology studies have identified gene mutations or defective genes. The majority of ND studies recognized the contribution of adverse environmental conditions, such as exposure to toxins, chemicals, nutritional deficits, social factors, drug abuse, and alcohol, leading to neurodegeneration and manifestation of pathology and behavioral defects. Many medications and therapies have been evaluated for these diseases, resulting in less than acceptable results [5,6]. Therefore, the necessity for novel treatments to improve symptoms and prevent ND progression is at an all-time high.

In the past decade and a half, ND has been the topic of an immense number of investigations. Much progress has been made, but for patients with ND disorder, these ailments remain overwhelming and deadly, emphasizing an urgent need to develop more effective treatments. Recent progress suggests that epigenetic modifications are highly relevant targets, and thus, they represent a potential site for pharmaceutical intervention. Aberrant epigenetic modifications related to ND disorders are starting to emerge and emphasize the great need to assess recent advancements in epigenetic mechanisms in ND disease research. We and others have recently discussed other mechanisms involved in severe ND disorders, including Alzheimer’s disease (AD), Huntington’s disease (HD), Parkinson’s disease (PD), and Amyotrophic lateral sclerosis (ALS) [7,8,9]. Therefore, the current review discusses the critical advancements in epigenetic changes, specifically histone methylation regulation in ND disorders.

## 2. Epigenetic Changes: Environmental Conditions and Agents That Induce ND

The term “epigenetic” denotes modification of gene expression without directly altering the DNA sequence [10]. These changes are heritable, and several of them are revocable [11]. These modifications occur at the molecular level and can be linked to several adverse factors, such as environmental cues, agents, and stress [12]. Although genetic factors critically influence phenotype outcomes, epigenetic changes provide a new level of intricacy to our perception of biology [13]. For many disorders, epigenetics contributes enormously to the disease’s onset and progression; this is an especially attractive possibility for diseases with unidentified etiology. Well-characterized epigenetic mechanisms include DNA methylation, microRNAs (miRNAs), and the posttranslational modification (PTM) of histone proteins [12,14,15]. We discuss recent advances related to histone protein methylation in several prominent neurodegenerative conditions and their role in the progression of ND in the following section.

## 3. PTM of DNA-Associated Histone Proteins

**Figure 1:**
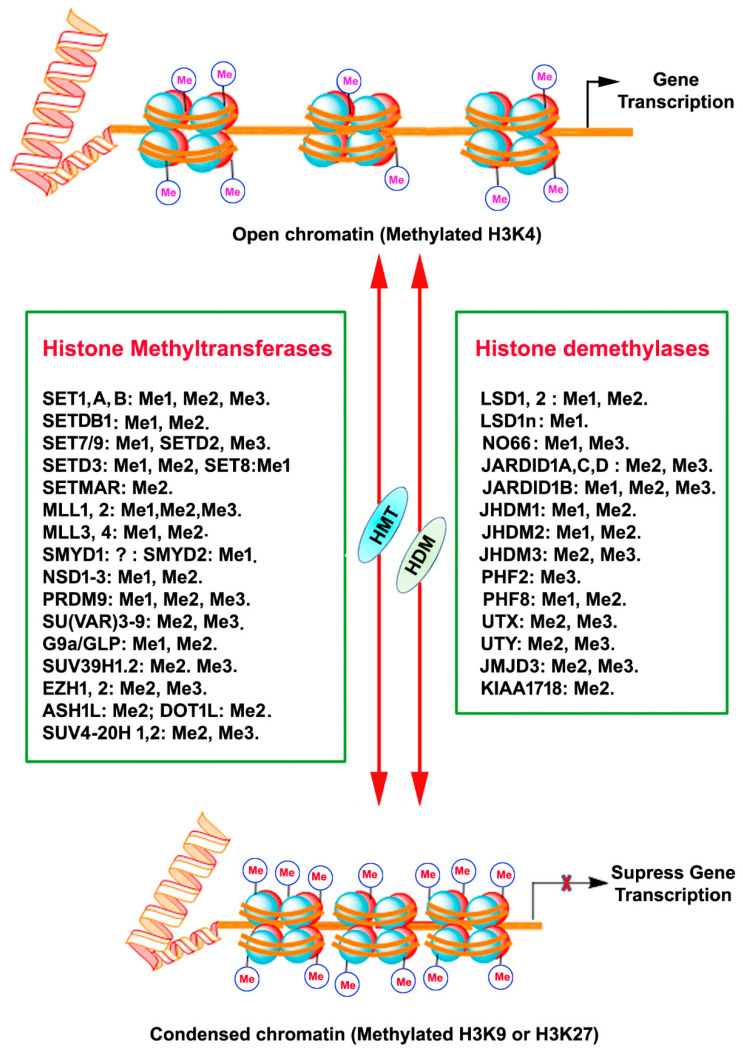
A schematic depiction of chromatin histone protein lysine methylation and demethylation by the mammalian histone methyltransferase (KMTs) and histone demethylase (KDMs) enzyme families. The HMT and HDM for each lysine methylation are also represented with their methylation/demethylation state specificities (Me1, monomethylation; Me2, dimethylation; Me3, trimethylation), X, inhibition.

Histones are a highly conserved set of nuclear proteins that assemble in an octamer composed of pairs of H2A, H2B, H3, and H4. Nuclear DNA is highly condensed and wrapped around these nuclear proteins, nucleosomes, in the form of chromatin to accommodate chromosomes in the nuclei [16]. The histone proteins’ N-terminal tail domain undergoes chemical modification through PTMs of each specific amino acid residues, including acetylation, methylation, phosphorylation, and sumoylation [17]. This unique chemical PTM impacts the chromatin structure and recruitment of DNA binding factors and allows for the relaxing or compacting of the chromatin structure around particular gene loci, resulting in the activation or repression of specific gene expression [17]. Histone methylation is one of the PTM that regulates gene expression. Adding or removing methyl groups on lysine (K) residues of histones by histone methyltransferases (HMT) or histone demethylases (HMD) alters the structure of chromatin to facilitate (relaxed chromatin) or prevent (condensed chromatin) access of transcription factor proteins to genomic DNA, thereby guiding gene expression or repression in a more complex manner [18,19] (Figure 1).

## 4. Histone Methylation

Histone methylation usually occurs at the arginine or lysine N-terminal region, which leads to the activation or suppression of gene expression. Each arginine residue in histones can be subjected to monomethylation and symmetric/asymmetric dimethylation. Likewise, each lysine residue can be mono-, di- and tri-methylated, and also nearby residues can form different methylation combinations [20,21,22,23]. Methylation of all the arginine residue is catalyzed by the family of arginine methyltransferase (PRMT) enzymes. PRMT1 monomethylates and asymmetrically dimethylates arginine residues, thus resulting in the activation of gene expression. PRMT2 promotes gene suppression through monomethylation and symmetric dimethylation of arginine residues. Moreover, the cofactor-associated arginine methyltransferase (CARM1) promotes gene activation through monomethylation and asymmetric dimethylation of arginine residues. HMTs that catalyze the methylation of lysine amino acids of histone proteins and other proteins are called lysine methyltransferases (KMTs) [24] for their extensive enzymatic activity and precise substrate specificity (Figure 1). All KMTs, except for KMT4, have a conserved Su (var)3–9, Enhancer of Zeste, Trithorax (SET) domain that presents catalytical activity [25]. The presence of a characteristic homologous sequence [24,26] has enabled the classification KMTs into well-defined subcategories. The KMTs that add the methyl group on H3K9, H3K27, and H4K20 are the main repressive KMTs, while the KMTs that add methyl group on H3K4, H3K14, and H3K36 are classified as activating KMTs [27]. One of the most highly investigated marks is H3 lysine (4) (H3K4) methylation, which is associated with active gene expression. Histone H3K4 methylation is catalyzed by a group (KMT2) of mixed lineage leukemia (MLL) proteins (SET1A, SET1B, MLL1, MLL2, MLL3, MLL4, and ASH1) [28,29,30]. H3K4me3 mark is found mainly in nucleosomes associated with the promoter regions of actively transcribed genes [31], while H3K4me2 is located in the gene bodies and enhancers associated with active genes [18]. H3K4me1 is found in enhancers, promoters, and at the 3′ end of active genes [32]. A family of methyltransferases (KMT1) (SUV39H1, SUV39H2, G9A/GLP and ESET/SETDB1; KMT8: RIZ1) catalyze histone H3K9 methylation and catalytical activity differ with catalyzing substrates and resulting products. Histone H3K9 trimethylation (H3K9me3) is catalyzed by SUV39 and results in a heterochromatin structure and transcriptional suppression [33]. Histone H3K9 dimethylation (H3K9me2) is catalyzed by G9A and results in a euchromatin structure and suppressed gene expression [34,35]. G9A-like protein (GLP) forms a hetero polypeptide complex with G9A, and, collectively, they catalyze the H3K9 dimethylation [36]. ESET/SETDB1 catalyzes the trimethylation of H3K9 [37], which results in the inhibition of gene expression. Among the alterations linked with transcription elongation, histone H3K36 trimethylation modification occurring at nucleosomes in the 3′ region of the transcription region of active genes [38]. H3K36me3 is catalyzed by KMT3 (SET2, SYMD2 and NSD1) enzymes [39,40,41]. Histone H3K79 di- and tri-methylation are catalyzed by DOT1L enzymes (KMT4) and are associated with gene expression activation [42]. Histone H4K20 mono- or tri-methylation is catalyzed by SET8 and SUV420H1/2 enzymes (KMT5), respectively, associated with gene inactivation [43]. The monomethylation of H3K27 often increases the expression of target genes, whereas the addition of three methyl groups at the same site usually suppresses gene transcription [44]. Histone H3K27me1 is catalyzed by ATXR5 and ATXR6 or TXR1 [45,46] and H3K27me3 is established by Enhancer of Zeste 2 (EZH2) of the polycomb repressive complex 2 (PRC2) [47,48,49].

## 5. Histone Demethylation

The amine oxidase containing lysine-specific demethylase 1 (LSD1/KDM1A) and lysine-specific demethylase 2 (LSD2/KDM1B) [50] remove mono- and dimethyl groups from histone H3K4 proteins [51]. Furthermore, KDM1B was shown to demethylate the mono- and di-methylated histone H3K9 [52]. However, amine oxidase demethylases are unable to catalyze the removal of trimethyl groups and are catalyzed by a set of Jumonji domain (Jmj) containing demethylases (JARID1a/KDM5A and JARID1b/KDM5B) [53,54]. A protein containing a Fe^2^^+^ dioxygenase Jumonji-C (JmjC) domain (KDM2 family proteins) was identified as a specific H3K36me1/2 demethylase (KDM2A and B), which does not demethylate H3K36me3 [55]. The KDM3 family of proteins were identified as the second group of JmjC histone demethylases (KDM3A/JHDM2A and KDM3B/JHDM2B) and were suggested to demethylate mono- and di-methylated H3K9, thereby promoting active gene expression [56]. The KDM4 family enzymes contain four histone demethylases (KDM4A–4D or JMJD2A–2D, respectively) and all exhibit di- and tri-demethylation of histone H3K9 and H3K36 proteins [57,58,59]. In addition to the JmjC domain, these demethylases have a conserved JmjN domain. All demethylases, except KDM4D, have tandem plant homeodomain (PHD) fingers and Tudor domains that facilitate recognizing specific histone methylation marks [60,61]. KDM5 (KDM5A–5D/JARID1A–1D) particularly catalyze the demethylation of histone H3K4me2/3 proteins [62,63,64,65,66]. These demethylase proteins are considered multi-domain proteins and comprise a mixture of JmjC/JmjN catalytic domains, an AT-rich interaction domain (ARID), a DNA-binding domain, a C5HC2 zinc finger and two–three PHD fingers. KDM5A and KDM5C PHD finger domains facilitate binding to methylated histone H3K4 or H3K9, respectively [63,67]. KDM6 family proteins include KDM6A/UTX and KDM6B/JMJD3 demethylases. These enzymes catalyze di- and trimethyl groups’ removal from histone H3K27me2/3 proteins [47,68,69,70,71] and cause gene transcription. The KDM7 (PHF2) family proteins contain three members of histone demethylases (KDM7A/JHDM1D, KDM7B/PHF8, and KDM7C/PHF2). This demethylase family removes mono- and dimethyl groups from histone H3K9me1/2 and H3K27me1/2 proteins [72,73,74,75,76,77,78,79]. Additionally, PHF8 demethylates histone H4K20me1 proteins [76,79,80]. KDM7 family proteins contain a PHD finger domain that assists recognition and binding to the histone H3K4me3 mark, leading to substrate specificity, genomic occupancy, and the regulation of target gene expression [73,78,81,82,83]. Removal of the trimethyl group from H3K27 is catalyzed by the Jmj-containing enzymes UTX/KDM6A and JMJD3/KDM6B [71]. As discussed below, studies have demonstrated that histone methylation and chromatin remodeling enzymes facilitate dynamic, complex tasks, such as synaptic plasticity, learning, and long-term memory formation [84] and may have a significant role in aging [85] and/or environmentally induced neurodegeneration that causes neuronal dysfunction. The HMTs and KDMs exhibit higher specificity and selectivity toward their lysine residues in each histone protein and are more stable, making them a highly appropriate and potential target for therapy.

## 6. Alzheimer’s Disease (AD)

AD is the most established neurodegenerative disorder in the world. Emerging findings indicate that epigenetic dysregulation of gene expression may play a significant role in aging and ND [86,87,88,89]. Although global accumulation or loss of histone methylation proteins and the subsequent alteration of the expression of many genes are shown in aging and cognitive functioning [32], the role of the diverse array of histone methylation, even in AD, has not been identified [86,90]. Additionally, a clinical study has shown modifications in H2B K108 and H4 arginine (R) 55 methylation in the frontal cortex from human donors with AD [91], suggesting that histone methylation may be a new potential therapeutic target to treat AD-related cognitive abnormalities. A postmortem AD brain study reported elevated levels of H3K9me2 proteins in the occipital cortex compared to nondemented and age-matched controls [92]. The inhibition of G9a/GLP catalytic activity by BIX 01294 prevents the Aβ oligomer-induced late-LTP and synaptic tagging and capture (STC) deficits by releasing the transcription repression of the Bdnf gene [93]. Moreover, BIX 01294 treatment in the hippocampus slices rescued Aβ oligomer-induced suppression of Bdnf gene expression [93]. Similarly, another study [94] has shown significantly elevated H3K9me2 and Emt1 (G9a) and Emt2 (GLP) in the PFC and HP from late-stage FAD mice and human patients with AD. These mice also exhibited reduced glutamate receptor transcription and many AD-like cognitive deficits. The elevated global H3K9me2 in FAD mice was also significantly enriched at the transcription start site regions of glutamate receptors (Gria2/GluA2 and Grin2b/NR2B genes), indicating that the loss of glutamate receptor transcription in AD is due to aberrant histone H3K9 dimethylation. Interestingly, pharmacological inhibition of H3K9me2 formation by GLP/G9a improved Gria2/GluA2, Grin2b/NR2B genes’ transcription, and that of additional genes (e.g., SHANK2) that are implicated in AD. These studies suggest that pharmacological inhibition (BIX 01294) of GLP/G9a normalizes multiple target genes and restores synaptic function and cognitive functioning in aged FAD mice [94]. Consistent with the above findings, exposure of mature human cortical neurons, derived from human embryonic stem cells, to Aβ significantly enhanced G9a, and inhibited AMPAR-mediated whole-cell current and excitatory postsynaptic current (EPSC) [95]. Further, the addition of BIX 01294 rescued EPSC in Aβ exposed human cortical neurons [95]. Interestingly, AD pathological hallmarks (hyperphosphorylated tau and Aβ plaques and cognitive deficits) exhibited by children and young adults in polluted cities showed reduced H3K9me2/me3 in postmortem prefrontal white matter [96]. Another more specific G9a/GLP inhibitor (UNC0642) was used to rescue 5XFAD cognition impairment and the H3K9me2 levels in the hippocampus [97]. The UNC0642 was successful in improving gene expression (Nuclear Factor erythroid-2-Related Factor 2 (NRF2), Heme oxygenase decycling 1 (Hmox1), Nerve growth factor (Ngf), Nerve growth factor inducible (Vgf), BDNF, and Synaptophysin (SYN)), protecting pathological changes (Reactive Oxygen Species, ROS; neuroinflammatory markers, such as Interleukin 6 (Il-6), Tumor necrosis factor-alpha (Tnf-α) gene expression, and Glial fibrillary acidic protein (GFAP), reduction in β-amyloid plaques) in 5XFAD mice [97]. A recent genetic study [98] of aging human brains has suggested that the strongly linked module with cognitive deficits is enriched with genes that regulate chromatin remodeling [98]. In contrast to the above-discussed findings, reduced H3K9me2 levels in the CA1 region of middle-aged and AD stages I-VI [99] were observed. A positive correlation between H3K4me3 marks and the long noncoding RNAs’ (lncRNA) gene expression level and a negative association between H3K27me3 marks and the lncRNA gene expression level was found in the CK-p25 AD model [100]. In contrast, decreased H3K4me3 with no change in H3K27me3 marks at the ANK1 gene locus in postmortem AD brains was reported [101]. Interestingly, an age-related increase in H3K27me3 was observed in neurofilament (NF)-labeled calretinin-positive interneurons [102]. However, amyloid plaque deposition and its sequelae failed to alter global H3K27me3 in NF-positive calretinin-labeled interneurons. The mislocalization of H3K4me3 between the nucleus vs. cytoplasm was observed in the medial temporal gyrus of the human postmortem AD brain [103]. The mislocalized cytoplasmic H3K4me3 was associated with pre-tangles and NFTs in late AD brains [103]. Similarly, a loss of nuclear H3K4me3 mark in hippocampal subregions but not cytoplasmic H3K4me3 mark was found in an AD animal (3×Tg) model, which develops plaques and NFTs [103]. Similarly, the H3K4m3 mark, as well as specific HMTs, such as KMT2A-D in the PFC nuclear fraction from human AD, was significantly enhanced without affecting H3K27me3 or H3K4me marks [104]. A similar increase in H3K4me3 and Kmt2a was also found in P301S transgenic Tau mice (PS19) [104]. These epigenetic advances provide robust experimental evidence using multiple AD models and suggest that restoring histone methylation’s homeostasis specifically mediated by G9a/GLP and KMT2A-D may perhaps be a potential therapeutic strategy to treat AD-related neurodegenerative disorders. Given the multifactorial characteristics of AD, targeting a single or a couple of abnormal genes is unlikely to prevent pathophysiological and behavioral abnormalities. Well-characterized histone methylation targeted pharmacotherapy in the future may offer the advantages of having broad, multifunctional actions and being able to target a network of genes essential for neuronal survival and function.

## 7. Huntington’s Disease (HD)

HD is a late-onset, autosomal progressive neurodegenerative disorder caused by the trinucleotide CAG repeat in the coding for glutamine (Q) in exon 1 of the Huntingtin (Htt) gene [105], leading to the motor, cognitive and psychiatric symptomatology [106,107]. Modification of the chromatin structure and deregulation of neuronal gene transcription are prominent features associated with the earliest stage of HD. Studies in recent decades using patients and multiple animal models of HD have identified histone modifications (acetylation, methylation, ubiquitylation, and phosphorylation) (see review [108]) and DNA modifications as significant epigenetic modifications that regulate gene expression in HD. The pharmacological approaches directed to correct some of those epigenetic changes have offered potential in treating HD (see review [109]). The following discussion presents the most recent advances in histone methylation-mediated epigenetics for potential HD disease interventions.

Enhanced H3K9me2 has been found in murine models of HD [110,111,112], suggesting that a gain of specific HMT or loss of HDM function appears to be associated with the pathogenic gene suppression observed in disease progression. SETDB1, a methyltransferase that explicitly targets the H3K9me3 [113], was enhanced in patients with HD and transgenic R6/2 HD mice [114]. Based on these observations, it was evident that neuronal levels of SETDB1 and H3K9me3 might be an indication of nucleosomal dysfunction in HD [115,116]. Similarly, monoallelic deletion of cyclic AMP response element-binding (CREB) protein (CBP) causes enhanced SETDB1 gene expression and H3K9 hypermethylation [117]. More importantly, enhanced H3K9me3 was found to promote the establishment of large constitutive heterochromatin domains and has been shown to promote both global and local [117,118] suppression of gene expression, including enrichment at the muscarinic acetylcholine (Ach) receptor 1 (CHRM1) gene promoter in HD striatal cells [117]. Consistent with these findings, the combined use of mithramycin, a clinically approved guanosine–cytosine-rich DNA binding antitumor antibiotic, and cystamine suppressed ESET expression by reducing H3K9 hypermethylation and significantly rescued behavioral and neuropathological phenotypes and extended survival by over 40% in R6/2 HD mice [117]. These observations suggest that epigenome regulation via H3K9me3 is vital for neuronal survival in HD and appears to be a potential treatment target in patients with HD.

Besides, studies have also shown a functional interaction of the Htt gene with protein arginine methyltransferase 5 (PRMT5), an enzyme mediating the dimethylation of arginine (R) of essential cellular proteins, including histones and spliceosomal proteins [119]. Htt has been shown to activate PRMT5 and reduce arginine dimethylation of histone H2A and H4 in primary cultured neurons and HD brains [119]. Consistent with this observation, the expression of PRMT5/methylosome protein 50 (MEP50) complexes, or the genetic ablation of the JMJD6 (H4R3Me2 demethylase), rescued the toxic effects of mutant Htt in primary cortical neurons [119], indicating that PRMT5 loss may be responsible, at least in part, for HD pathogenesis. It was shown that Htt null embryos exhibited impaired PRC2 [120], a methyltransferase complex containing Ezh2 that adds the trimethyl group to H3K27 and maintains the gene expression pattern by regulating the chromatin structure in mitotic and postmitotic cells in the brain [121,122]. Similarly, the lack of Htt in embryos reduced histone H3K27me3 and impaired homeobox (Hox) gene expression and trophoblast giant cell differentiation, causing paternal X chromosome inactivation [120]. Consistent with the results, genome-wide analysis in *Htt* WT and targeted inactivation of both copies of *Htt* (dKO) genotypes suggested that the loss of Htt triggered a significant reduction in the total number of H3K27me3-marked promoters [123]. These findings significantly imply that huntingtin is required to retain the H3K27 trimethyl group added by PRC2 efficiently. The effect of huntingtin on facilitating the enrichment of H3K27me3 could be achieved through the physical interaction between full-length huntingtin and the PRC2 complex [120], such as the Ezh2 or Suz12 or Utx (demethylates H3K27me3) [68,124].

The additional function of PRC2 in HD was supported by H3K27me3 ChIP-sequencing in neuronal chromatin obtained from the HD postmortem PFC and non-neurologic controls. The findings suggest the loss of neuronal PRC2-H3K27me3 sites and the upregulation of some PRC2 target genes associated mainly with Hox gene clusters and developmentally related proteins in the HD-affected human brain [125,126,127,128]. Remarkably, loss of PRC2 levels in adult neurons was associated with the derepression of selected PRC2 target genes, followed by loss of neuronal functions and survival, therefore further strengthening the observation that persistent dysregulation of PRC2, in addition to other H3K27me3-controlling enzymes, may cause systemic neurodegeneration in HD [129]. Nevertheless, the presence of an expanded CAG tract is not only related to changes in the PRC2 pattern and changes in histone H3K27me3 enrichment, causing reduced RNA expression [123]. This observation emphasizes that histone-modifying enzymes and chromatin remodeling factors do not function as a single molecule but act as a supermolecular complex to control gene transcription in a coordinated but complementary manner (repression and activation) [130].

Notably, the diminished enrichment of H3K4me3, a mark of active gene transcription, has been associated with impaired transcription of target genes (Bdnf, Penk1, Drd2) in both human HD postmortem brains as well as the cortex and striatum of R6/2 mice [131]. Reduced H3K4me3 enrichment was observed as the RE-1 silencing transcription factor/neuron-restrictive silencer factor (REST/NRSF) promoter II [131], therefore indicating that impaired transcription might be a result of changes in chromatin structure at the REST binding site on the *BDNF* gene locus [131]. These observations are in line with previous results showing changed REST and BDNF signaling in HD [132,133]. Furthermore, the knockdown of JARID1C by ShRNA (demethylates H3K4me3) caused the upregulation of Bdnf gene expression in primary cortical neurons derived from the BACHD mouse HD model and improved the health of these neurons, indicating neuroprotection against HD in this model. Similarly, enhanced JARID1C was also observed in HD *Htt* (Q150) knock-in mice [134]. Notably, JARID1C, which has been shown to interact with REST [65]), and the NRSE motif are strongly enriched near sites of reduced H3K4me3 in R6/7 mice [131]. A similarly reduced function of H3K4me3 at the genome-wide level in HD postmortem PFC tissues [125,135] was reported. These findings indicate that chromatin-remodeling enzymes, including SETDB1, PRMT5, Ezh2, JARID1C, may be potential therapeutic targets for HD treatment [131], further strengthening the observation that repressive neuronal chromatin mediated by histone methylation may have a crucial role in HD pathophysiology, including ND.

## 8. Parkinson’s Disease (PD)

PD is the second most predominant neurodegenerative disorder in the world after AD [136]. The most significant pathology of PD is the demise of dopaminergic neurons in the substantia nigra with Lewy bodies (aggregated alpha-synuclein and ubiquitin-protein and damaged nerve cells as cytoplasmic inclusions) [137,138,139]. PD is characterized by motor symptoms (bradykinesia, tremor, postural instability, and rigidity) and nonmotor symptoms (cognitive issues, autonomic dysfunction, and REM sleep behavior disorder) [137]. However, the mechanisms involved in PD pathogenesis have not been fully identified. The specific loss of dopaminergic neurons in PD has been believed to be a consequence of complex interactions between genetic and environmental factors. Nevertheless, the characteristics of the relationship between the two significant changes remain to be established. A growing body of studies have begun to support epigenetic events, such as DNA methylation and histone modification in PD progression [140,141,142].

A mounting number of studies overwhelmingly suggest the critical function of the histone modification and PD-associated α-synuclein coding gene SNCA expression [143], meaning that histone methylation also may have a crucial role in the regulation of the SNCA gene. Interestingly, overexpression of α-SYN in flies and neuronal cells, such as SH-SY5Y enhanced G9a, H3K9me1, and H3K9me2 levels and H3K9me2-target genes (L1cam, Snap25) eventually lead to impaired synaptic activity [144]. In contrast, H3K4me3 was significantly enriched at the *SNCA* promoter region in postmortem brain samples from patients with PD and matched controls [145]. Similarly, using dead Cas9-Suntag system-mediated locus-specific approaches, the reduction in H3K4me3 from the *SNCA* promoter reduced α-synuclein levels in neuronal cell lines and PD-derived induced pluripotent stem cell lines (iPSCs) [145]. Significant H3K4me3 enrichment was observed at the *SNCA* promoter in the neuronal nuclei (NeuN) of positive neurons of substantia nigra (SN) tissue samples from PD patients. Interestingly, significant reductions in H3K4me3 and H3K27me3 marks were observed in SH–SY5Y cells treated with the neurotoxin 6-hydroxydopamine (6-OHDA) [146]. In the same model, pretreatment with GSK-J4, a potent inhibitor of KDM6A/B and KDM5B/C (demethylates H3K27me3/me2 and H3K4me3/me2 respectively) [147,148], significantly prevented H3K4me3 and H3K27me3 marks’ decrease [146]. In contrast to the cell culture model, increased H3K27me3 levels were found in PD patients’ brains [145], indicating the possible role of PRC2 in vivo PD models. These findings indicate that abnormal histone methylation, such as H3K4me3 (gene activation) and H3K27me3 (gene suppression), control gene expression (e.g., α-synuclein) in SN neurons from patients with PD. Because specific histone methylation is one of the central regulators of gene expression, future investigation is warranted to understand further how specific histone methylation of the gene s, including the *SNCA* gene, directly regulates the accessibility of transcription factors that can access gene regulatory regions. Finally, there are still undiscovered histone methylation marks that may regulate synaptic and pathological abnormalities found in PD. Those may also have a considerable impact on regulating chromatin structure and overall effects on gene transcription in PD and should be of future interest.

## 9. Amyotrophic Lateral Sclerosis (ALS)

ALS is a fatal ND distinguished by the selective loss of both upper and lower motor neurons [149]. There is substantial variability in the ALS phenotype regarding age and location of onset, the relative degree of upper and lower motor neuronal loss, and degree of progression [150]. Because motor neurons in the spinal cord, brain, and brainstem deteriorate, skeletal muscular atrophy extends through the patient [151]. Diagnosis is inadequate and quality of life is drastically low, as most patients’ survival is somewhat short with a median of 2 to 4 years of diagnosis [152]. ALS is classified as familial and sporadic types. Familial ALS denotes about 10% of all cases and the disease could be assigned to a specific gene mutation happening in families. Sporadic ALS represents the remaining 90% of all cases and there is no family history of the disease found. However, both familial and sporadic cases are clinically indistinguishable and contribute to a similar pathology [153]. Despite substantial research efforts over the last two decades, no biomarkers or effective therapeutics have been discovered to rescue, slow down, or stop neuronal loss in patients. Several ALS-associated genes are identified; nevertheless, genetic mutations do not exclusively account for neurodegeneration, and they fail to show the existence of a large number of idiopathic cases. Strikingly, sporadic ALS has been shown to be associated with many environmental insults, such as infectious agents, heavy metal toxicity and exposure to fertilizers and pesticides [154]. Many of these environmental agents are suggested to play a role in molecular mechanisms that facilitate epigenetic changes. These observations emphasize the role of genetic variation, and environmental exposure significantly contributes to ALS. It is also expected that environmental exposures influence epigenetic mechanisms that reversibly control gene expression and may contribute to the onset and progression of ALS [155].

Few epigenetic changes were reported related to ALS before discovering noncoding GGGGCC hexanucleotide repeat (HRE) in the gene C9ORF72 that is strongly associated with ALS disease [156,157], which was previously reported to be conclusively associated with chromosome 9p. This same repeat expansion was identified in the majority of transactive response DNA binding proteins with M_r_ 43 kD (TDP-43)-based pathology [157]. Because the alteration of epigenetic marks has been reported in many repeat expansion disorders [158], the observations of an HRE in *C9orf72* exposed the likelihood that epigenetic changes might also involve ALS pathology. The studies of epigenetic changes in ALS are still in infancy and much remains to be studied. Because other epigenetic modifications in ALS have been recently reviewed [153,159], we discuss the recent development of histone methylation in ALS in the following sections.

The reduced *C9orf72* mRNA in the frontal cortices and cerebella of c9FTD/ALS patients is associated with increased binding of *C9orf72* to H3K9me3, H3K27me3, and H4K20me3 marks [160], associated with heterochromatin and transcriptional repression [27]. Further, exposing repeat carrier-derived fibroblasts to 5-aza-2-deoxycytidine, a DNA and histone demethylating agent, not only reduced *C9orf72* binding to trimethylated histones (H3K9me3, H3K27me3, and H4K20me3), but also enhanced *C9orf72* gene expression [160]. In yeast models of ALS, it was shown that fused in sarcoma (FUS), a DNA/RNA binding protein, overexpression is associated with decreased levels of asymmetric dimethylation on arginine 3 on histone H4 (H4R3me2asym). Similarly, overexpression of TDP-43 and FUS resulted in reduced H3K36me3. H34Rme2asym promotes active gene expression [161], whereas H3K36me3 inhibits gene transcription by functioning as a docking site for HDACs thereby promoting histone deacetylation [162,163]. In a FUS^R521C^ mouse model of ALS, overexpression of PRMT1 rescues neurite growth associated with oxidative stress [164] through a mechanism that involves PRMT1 interacting with FUS leading to a stable complex of FUS/PRMT1/Nd1-L causing inhibition of PRMT1 activity. Nd1-L is an actin-stabilizing protein, and its expression is reduced in the FUS^R521C^ mouse model of ALS [164]. Consistent with these studies, loss of PRMT1 function due to FUS interaction caused a reduction in H4R3me2asym, leading to reduced acetylation of histone H3 on lysine 9 (H3K9ac) and lysine 14 (H3K14ac), eventually causing transcriptional suppression [165]. TDP-43 and FUS are known to cause protein aggregation and the formation of cytoplasmic inclusions in motor neurons and protein aggregation found in both familial and sporadic forms of ALS, as 97% of patients exhibit TDP-43 inclusions irrespective of whether they show mutations in this protein [166]. It is, therefore, far clear that histone methylation discussed above significantly contributes to the gene regulation in ALS. Enhanced H3K9me3 marks were found within the promoter of the human C9ORF72 transgene in the brain of C9-BAC mice by week 7, suggesting that partial epigenetic repression of the C9ORF72 gene locus occurs in the first postnatal weeks of life [167]. There was a correlation with a global decrease in H3K9me3 mark in astrocytes and neurons of spinal cord, motor cortex, hippocampus brain regions, neurodegeneration, and memory deficit in C9ALS/FTD BAC mice [167]. Similarly, decreased H3K9me3 mark was associated with the pathological findings in C9orf72 BAC transgenic mice [168], suggesting the contribution of SUV39H1/2, a histone lysine methyltransferase or Kdm4d demethylase (demethylates H3K9me3) responsible for H3K9me3 mark, in the pathophysiology of ALS. Based on the above-discussed observations, it is clear that more than one disrupted histone methylation mediated transcription underlies the synaptic, cognitive and pathological hallmarks in ALS. Therefore, identifying specific HTM or KDM and targeting histone methylation enzymes may represent a novel therapeutic strategy for this prevalent neurodegenerative disorder.

Collectively, the findings discussed above (Table 1) suggest a more significant impact of histone methylation-mediated transcription of genes in AD, HD, PD, and ALS. In the future, advancement in specific tissue and cell type-specific mechanisms would help to develop targeted, safer drugs for histone methylation for therapeutic intervention of neurodegenerative disorders.

## 10. Alcohol Use Disorders (AUD)

Because alcohol is a neurotoxic agent, its use has been associated with neurodegeneration [169,170,171,172,173,174] and shown to cause various neural diseases, including fetal alcohol syndrome (FAS)/fetal alcohol spectrum disorders (FASD), cerebellar degeneracy, alcohol addiction, and alcoholic dementia. However, the molecular underpinnings of alcohol use-induced neuronal diseases are limited despite several studies. Studies in recent decades using patients and multiple animal models of alcohol exposure have discovered histone (acetylation and methylation) and DNA modifications as significant epigenetic changes that regulate gene expression in alcohol use disorders (AUDs). While histone acetylation and DNA methylation roles in AUD [14,175,176,177,178] have been reviewed recently, this article presents the most recent advances in histone methylation in AUD, including developmental alcohol.

Investigation on histone methylation in AUD is in infancy, and studies are beginning to explore the function of each different histone methylation regulated gene expression. As discussed earlier, histone methylation could regulate gene repression (e.g., H3K27me3) or activation (e.g., H3K4me3). Postmortem brains of alcoholic individuals [179] exhibited increased H3K4me3 at expressed, non-expressed, and non-genic gene regions. Many genes showed high levels of H3K4me3 marks at the promoters presenting low levels of expression. These observations suggest that in addition to the H3K4me3 mark, other histone methylation marks may play a role in AUD [179]. Similarly, the increased global H3K4me3 mark and its associated GC-rich gene (*GIPC1*, *BCL2L1*, and *UBE1)* expressions were observed in the PFC and HP of postmortem alcoholic brain tissues [180]. H3K27me3 was enhanced via recruitment of EZH2 at the BDNF and ARC loci in the amygdala of the alcoholic postmortem brain, with the corresponding decrease in Arc and BDNF expression in the early-onset AUD postmortem brain amygdala [181]. Consistent with the above observations, increased KDM6B, which regulates H3K27me3 levels, was found in individuals with AUD in the anterior cingulate cortex [182]. These limited studies pinpoint the participation of both H3K4me3 and H3K27me3 marks’ mediated gene regulation in human AUD’s pathophysiology.

Similar observations were also observed in animal studies decreased H3K27me3 at the Mt1 gene promoter and increased global H3K4me3 levels at the Mt2 gene promoter in the cerebral cortex were observed when mice were exposed to acute alcohol for 6 h [183]. Increased prodynorphin (PDYN) and pronociceptin (PNOC) gene expression in the amygdala of one-day ethanol-treated rats was associated with reduced H3K27me3 levels and increased H3K9me2 levels [184,185]. Chronic ethanol intake caused increased enrichment of H3K4me3 at BDNF exon II and reductions in H3K4me3 enrichment at III BDNF exon VIII, and these epigenetic changes were associated with their expression in the HP [186]. Chronic intermittent ethanol (CIE) treatment caused increased N-methyl-D-aspartate (NMDA) receptor 2B subunit (NR2B) expression, perhaps by decreasing the enrichment of H3K9me2 at the NR2B gene promoter [187]. Interestingly, CIE significantly reduced the expression of nine genes coding for histone methyltransferase (HMT) family enzymes (Setd1a, Setd1b, Setdb2, Suv39 h1, Setd4, Setd6, Setdb1, Prmt6, and G9a) [187]. Enrichment of H3K4me2 at cFos, Cdk5, and FosB genes promoter regions in the PFC of adolescent intermittent ethanol (AIE) exposed brain was found [188]. AIE exposure decreased the mRNA and protein levels of lysine demethylase 1 (LSD1) and increased global H3K9me2 in the rat central and medial nucleus of the amygdala (CeA and MeA) [189]. Similarly, functional loss of Lsd1+8a (a neuron-specific splice variant of LSD1) in these mice rescued the increased enrichment of H3K9me2 at the BDNF exon IV gene promoter region in the amygdala caused by AIE [189]. Histone methyltransferase PR domain containing 2, with ZNF domain (PRDM2) protein, an enzyme responsible for mono-methylation of H3K9 (H3K9me1), was reduced, followed by reduced H3K9me1, in the adolescent PFC of alcohol-dependent rodents [190]. Inhibition of PRDM2 via shRNA increased alcohol self-administration [190]. A reduction in myelin-related genes (*Mag*, *Mbp*, *Mobp*, and *Plp)* and H3K36me levels was found in AIE adolescent PFC [191]. A recent study using a genome-wide analysis identified a global decrease in H3K4me3 peaks and enhanced H3K27me3 peaks in ethanol-withdrawn mice compared to controls, which were correlated with persistent reductions in gene expression [192]. In the same study, pathway analysis of genes associated with changes in H3K4me3 and H3K27me3 levels uncovered enrichment of genes that are critical for proteoglycan and calcium signaling pathways [192]. Consistent with the above studies, reduced KDM6B expression and reduced occupancy of KDM6B and H3K27me3 at the synaptic activity response element (SARE) site and promoter of the *Arc* gene, enabling increased negative elongation factor (NELF) binding to the *Arc* promoter was observed in AIE adult rats. These events inhibited A*rc* expression in the amygdala. Inhibition of *Kdm6b* or *Arc* eRNA expression in the CeA significantly increased H3K27me3 occupancy at the Arc SARE site and recapitulated the molecular and chromatin phenotypes of AIE [193]. Alcohol vapor exposure for 72h in WP and WSP mice reduced *Smyd3* (di- and trimethylates H3K4), *Setdb1* (trimethylates H3K9) and Setd6 (mono-methylates H2AZK8) gene expression. This paradigm also increased *Setd7* (mono-methylates H3K4), *Setd3* (methylates H3K4 and H3K36), *Ash1l* (methylates H3K36) [194]. These findings collectively suggest novel suppressive chromatin mediated by H3K9me2, H3K4me3, and H3K27me3 marks mechanisms that participate in AUD and may have a significant therapeutic utility to treat AUDs (Table 2).

Alcohol exposure during early development has been shown to induce neuronal loss at different stages of brain development, leading to neuronal dysfunction at the adult stage. In addition to histone acetylation and DNA methylation changes, histone methylation changes have been shown in many developmental alcohol studies. GD 7 acute ethanol exposure increased H3K9me2, decreased H3K27me3 and altered G9a, Setdb1, Kdm1a, Kdm4c, Uhrf1 and Ezh2 mRNA levels [195]. Reduced expression of the Pomc gene and β-endorphin protein in the arcuate (ARC) nucleus of the hypothalamic region of prenatal alcohol-exposed male offspring and correlated with reduced staining of H3K4me2,3 and increased H3K9me2 [196,197]. These epigenetic changes were associated with impaired hypothalamus function, such as stress axis responsiveness. Altered expression of three genes coding for HMT family enzymes (Set7/9, Setdb1, and G9a) was observed in PAE offsprings [197]. Similarly, enhanced global H3K9me2 levels, G9a gene [198], and protein expression in the neocortex and HP tissues [35,205] were found in the postnatal ethanol exposure (PEE) model, which is equivalent to third-trimester human pregnancy. Interestingly, the use of a G9a (Bix 01294) inhibitor rescued both ND in neonatal mice and Arc gene, synaptic plasticity, learning, and memory defects in adult mice [34,198,199,205]. Consistent with the above observations, increased H3K9me2 and G9a at Rac1 [200] and Arc [205], whereas reduced H3K9me2 at cannabinoid receptor 1 (Cnr1) [201] gene promoters were observed in PEE adult mice HP and Nc regions. Also, increased enrichment of H3K4me3 at the promoter region of the Slc17a6 gene encoding VGLUT2 protein was identified in PAE brains [202]. Similarly, acute alcohol exposure at gestational day 7 (GD 7) in mice significantly enhanced H3K9me2 levels and reduced H3K27me3 levels at GD 17, and these histone methylation changes were strongly associated with craniofacial and central nervous system (CNS) abnormalities [195]. Increased global levels of H3K4me3 and expression of lysine N-methyltransferase 2 (Kmt2e) that catalyzes H3K4 trimethylation in the PEE neonatal brain cortex and cerebellum [204] was reported. In contrast to the above study, decreased temporal lobe H3K4me3 levels in select regions were observed in autopsied fetuses and infants [203]. In macaques, reduced H3K36me3 levels were found in the dentate gyrus (DG) region and ependyma [203]. These studies collectively suggest that an epigenetic signature mediated by H3K4me2,3, H3K9me2, H3K27me3 and H3K36me3 marks induced during early developmental ethanol exposure insults continues long after the window of exposure and is generally associated with changes in a repressive chromatin structure in human and animals studies (Table 2). Persistent changes associated with repressive chromatin structure associated with important synaptic plasticity genes such as Arc, Egr 1, Rac1, Pomc and Slc17a6 genes underly the utility of rodent models to evaluate epigenetic mechanisms of early ethanol use disorders such as FASD.

**Figure 2:**
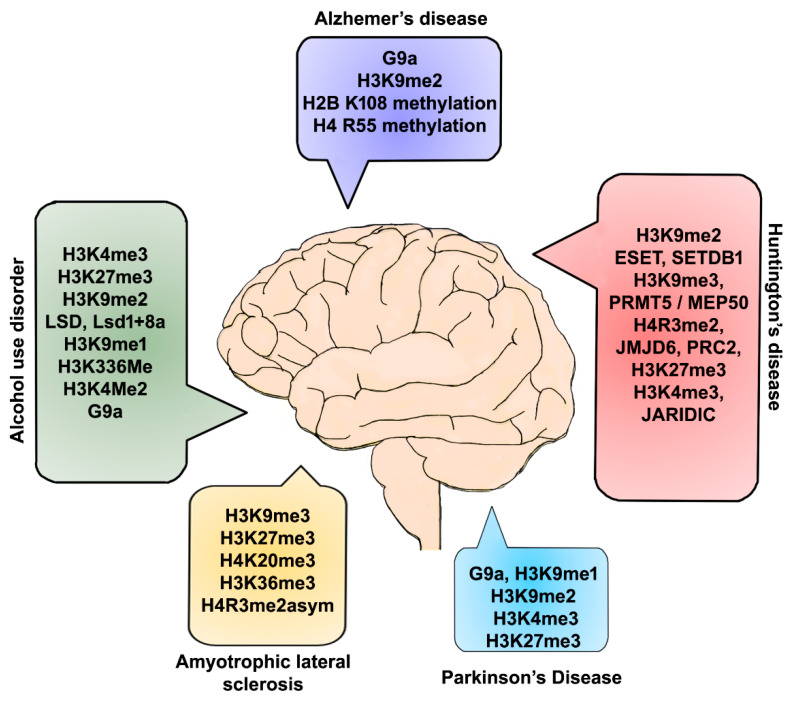
Graphical summary of histone methylation-specific epigenetic defects in ND disorders. Neurodegeneration-inducing conditions have been shown to affect DNA-associated histone methylation via specific KMTs and KDMs, followed by several changes in gene and protein expression that are important for cognitive and other ND-related brain pathologies.

In summary, with the available findings, it is clear that suppressive chromatin mediated by various histone marks regulated by a dynamic balance between specific HMT and HMD significantly contributes to the neurodegeneration and progression of disease pathology (Figure 2). Given the strong link between the dysregulation of gene expression and several neurodegenerative disorders, understanding the specific epigenetic pathway responsible for each of these disorders’ pathophysiology is an ongoing aim of several laboratories. Histone methylation has been linked to the suppression of gene expression in both brain development and cognitive behavior in adults, yet the underlying mechanisms remain obscure. Beyond its direct therapeutic value, studies are looking to understand each enzyme’s role that regulates the histone methylation status to develop specific analyses and mechanisms in neurodegenerative disorders. The identification and characterization of novel suppressive chromatin, in detail, are vital for understanding several neurodegenerative diseases and developing novel therapeutics and are essential tasks for the future. Detailed characterization of these events in the future will substantially contribute to the development of potential therapeutic agents to treat devastating ND disorders, even though these disorders differ in their fundamental causes and pathophysiology.

## Figures and Tables

**Table 1:** Histone methylation defects in neurodegenerative disorders.

Diseases	Enzyme	Histones	Model	Genetic Background	Genes	Brain Region	Effect	References
AD		H2BK108me	Human			PFC	↓	[91]
		H4R55me	Human			PFC	↓	[91]
		H3K9me2	Human			OC	↑	[92]
		H3K9me2/ me3	Human			CA1	↓	[99]
		H3K9me2	FAD mice	C57BL/6J		PFC HP	↑	[94]
		H3K9me2	Human			PFC	↑	[94]
	EMT1 (GLP) EMT2 (G9a)		FAD mice	C57BL/6J		PFC HP	↑	[94]
	G9a/GLP		Human			PFC	↑	[94]
		H3K9me2	FAD mice	C57BL/6J	Gria2/ GluA2	PFC HC	↑	[94]
		H3K9me2	FAD mice	C57BL/6J	Grin2b/ NR2B	PFC HC	↑	[94]
		H3K9me2	FAD mice	C57BL/6J	Shank2	PFC HC	↑	[94]
	G9a		Aβ-treated human stem cell-derived neurons.			Cell Cultures	↑	[95]
		H3K9me2/ me3	Human			PFC	↓	[96]
		H3K4me3	CK-p25 AD		lncRNA		↑	[100]
		H3K27me3	CK-p25 AD		lncRNA		↓	[100]
		H3K4me3	Human		ANK1			[101]
		H3K4me3	Human			Nucleus	↓	[103]
		H3K4me3	Human			Cytoplasm	↑	[103]
		H3K4me3	3×Tg mice			Nucleus	↓	[103]
		H3K4me3	Human			PFC-Nucleus	↑	[104]
		H3K4me3	PS19 mice			PFC-Nucleus	↑	[104]
		KMT2A-D	Human			PFC-Nucleus	↑	[104]
		Kmt2a-d	PS19			PFC-Nucleus	↑	[104]
HD		H3K9me2	R6/2 mice	C57BL/6J		ST	↑	[110,111,112]
	SETDB1	H3K9me3	Human R6/2 mice	C57BL/6J		NC, ST, CuN	↑	[113]
		H3K9me3	ST*Hdh* Q7/7 and ST*Hdh* Q111/111 cells				↑	[117]
		H3K9me3	Human, R6/7 mice, HD cell lines		Chrm1, Pdgfb, Inpp5j, Hrh1, Irf6, Eya1, and Kif5c		↑	[117]
	PRMT5	H2A/H4 sDMA of R3	mutantHtt fragment			In vitro activity	↓	[119]
		H2A/H4 sDMA of R3	mutantHtt fragment			Transfected primary neurons	↓	[119]
		PRC2	*Hdh^ex4/5^ Embryos*				↑	[120]
		H3K27me3	*Hdh^ex4/5^ Embryos*				↓	[120]
		H3K27me3	*Hdh*^ex4/5/ex4/5^ ESC and NPC			Bivalent loci	↓	[123]
		H3K4me3	HD and R6/2 mouse			Bdnf, Penk1, Drd2	↓	[131]
		H3K4me3	HD and R6/2 mouse			REST/NRSF	↓	[131]
	* Jarid1c *	-	HD and R6/2 mouse				↑	[131]
	*Jarid1c*	-	*Htt* (Q150) knockin mice				↑	[134]
PD		H3K9me1 H3K9me2	Transgenic *Drosophila* and inducible SH-SY5Y neuroblastoma cells				↑	[144]
	G9a	H3K9me2	αS-induced SH-SY5Y cells			L1cam, Snap25	↑	[144]
		H3K4me3	Human PD			Snca	↑	[145]
		H3K27me3	Human PD			-	↑	[145]
		H3K27me3	SH-SY5Y cells+ 6-OHDA			-	↓	[146]
		H3K4me3	SH-SY5Y cells+ 6-OHDA			-	↓	[146]
		H3K27me3	SH-SY5Y cells+ 6-OHDA			-	↓	[146]
		H3K4me3	SH-SY5Y cells+ 6-OHDA			-	↓	[146]
		H3K27me3	SH-SY5Y cells+ 6-OHDA +GSK-J4			-	↑	[146]
		H3K4me3	SH-SY5Y cells+ 6-OHDA++GSK-J4			-	↑	[146]
		H3K27me3	SH-SY5Y cells+ 6-OHDA++GSK-J4			-	↑	[146]
		H3K4me3	SH-SY5Y cells+ 6-OHDA++GSK-J4			-	↑	[146]
ALS		H3K9me3, H3K27me3 H4K20me3	Human ALS (c9FTD/ALS)			*C9orf72*	↑	[160]
		H4R3me2asym	yeast models of ALS (over expression of FUS)			-	↓	[160]
		H3K36me3	yeast models of ALS (over expression of TDP-43)			-	↓	[160]
	PMRT1		FUS^R521C^ ALS model (Overexpression of PMRT1)			-	↓	[160]
		H4R3me2asym	FUS^R521C^ ALS model (loss of PMRT1 function)			-	↓	[160]
		H3K9me3	C9ALS/FTD BAC mice			*C9 or f72*	↑	[167]
		H3K9me3	C9ALS/FTD BAC mice			*SC, NC, HP*	↑	[167]
		H3K9me3	C9ALS/FTD BAC mice			Cultured Astrocytes and neurons	↓	[168]

OC—occipital cortex, ST—striatum, NC—neocortex, CuN—caudate nucleus, Htt—huntingtin; PRMT5—protein arginine methyltransferase 5; *Hdh^ex4/5^*—huntingtin null *Hdh^ex4/5^* homozygote; HD—Huntington’s disease; AD—Alzheimer’s disease; PD—Parkinson’s disease; ALS—Amyotrophic lateral sclerosis; *PFC*—prefrontal cortex; *HP*—Hippocampus; EMT1 (GLP) —Euchromatic Histone Lysine Methyltransferase 1; EMT2 (G9a)—Euchromatic Histone Lysine Methyltransferase; SETDB1—Set domain bifurcated; PRMT5—Protein Arginine Methyltransferase 5; *JARID1C*—Jumonji AT-rich interactive domain 1C; PMRT1—Protein arginine methyltransferase-1; H3K9me1—Histone3 lysine 9 monomthylation; H3K9me2—Histone3 lysine 9 dimthylation; H3K9me3—Histone3 lysine 9 trimthylation; H4R55me—Histone 4 arginine 55 monomethylation (H4R55me); H2BK108me—Histone 2B lysine 108 monomethylation; H2A sDMA—Histone 2A symmetric dimethylarginine; H4sDMA—Histone 4 symmetric dimethylarginine; PRC2—Polycomb repressive complex 2; H3K27me3—Histone 3 lysine 27 trimethylation, H3K4Me3—Histone 3 lysine 4 trimethylation; H4K20me3—Histone 4 lysine 20 trimethylation; H4R3me2 asym—Histone 4 arginine 3 dimethylation asymmetric; H3K36Me3—Histone 3 lysine 39 trimethylation; L1cam—L1 Cell Adhesion Molecule; Snap25—Synaptosomal-Associated Protein, 25kDa; Snca—Synuclein Alpha; C90rf72—chromosome 9 open reading frame 72; SC—superior colliculus; chrm1, Cholinergic Receptor Muscarinic 1; pdgfb—Platelet Derived Growth Factor Subunit B; Inpp5j—Inositol Polyphosphate-5-Phosphatase J; Hrh1—Histamine Receptor H1; Irf6—Interferon Regulatory Factor 6; Eya 1—Eyes absent homolog 1; Kif5C—Kinesin Family Member 5C; Bdnf—Brain-derived neurotrophic factor; Penk1—Proenkephalin1; *Drd2*, Dopamine Receptor D2; REST/NRSF—RE1-Silencing Transcription factor/Neuron-Restrictive Silencer Factor; 6-OHDA—6-hydroxydopamine; -, no change; ↑, increased; ↓, reduced.

**Table 2:** Influence of alcohol exposure on brain histone methylation dynamics.

Alcohol Exposure	Tissue Examined	Effects
Postmortem Human alcoholic brain	PFC	Increased Global H3K4me3 at *GIPC1*, *BCL2L1*, and *UBE1 genes* [180].
Postmortem Human alcoholic brain	HP	Increased H3K4me3 at expressed, non-expressed, and non-genic gene regions [179].
Postmortem Human alcoholic brain	Amygdala	Increased recruitment of Ezh2, which regulates H3K27me3 levels, at BDNF and ARC gene locus in early-onset AUD group [181].
Postmortem Human alcoholic brain	ACC	Increased KDM6b [182] that regulates H3K27me3 levels.
Acute alcohol in mice	CCx and HP	Decreased H3K27me3 at Mt1 gene promoter; Increased H3K4me3 at Mt2 gene promoter [183].
Acute alcohol in rats	Amygdala	Decreased H3K27me3 levels; Increased H3K9me2 [184,185].
Chronic ethanol (free choice paradigm) in mice	HP	Increased H3K27me3 at Bdnf PII and PIII. Decreased H3K4me3 at Bdnf PVIII [186].
Chronic intermittent ethanol (CIE) in mice cortical neurons	Cortical	Decreased H3K9me2; Decreased Setd1a, Setd1b, Setdb2, Suv39 h1, Setd4, Setd6, Setdb1, Prmt6, and G9a; Decreased G9a, Suv39h1 at the NR2B gene promoter [187].
Binge-like ethanol in adolescent rats	PFC	Increased H3K4me2 at cFos, Cdk5, and FosB genes promoters [188]
Intermittent alcohol exposure in adolescent rats	Amygdala	Decreased LSD1 and LSD1+8A in CeA and MeA; Increased H3K9me2 in CeA and MeA [189].
CIA in rats	dmPFC	Decreased PRDM2 expession; Inhibition of PRDM2 in dmPFC via shRNA increased alcohol self-administration [190].
Intermittent alcohol exposure in adolescent mice	PFC	Reduced H3K36m1, me2 and me3 levels [191].
Alcohol vapor exposure in WSR mice	PFC	Increased H3K27me3 Reduced H3K4me3
Alcohol vapor exposure for 72h in WP and WSP mice	PFC	Reduced *Smyd3,* which di- and trimethylates H3K4. Reduced *Setdb1,* which trimethylates *H3K9.* Reduced *Setd6,* which mono-methylates the lysine 8 on the histone variant H2AZ (H2AZK8me1). Increased *Setd7*, which mono-methylates H3K4; Increased *Setd3*, which methylates H3K4 and H3K36; Increased *Ash1l*, which methylates H3K36 [192].
Intermittent alcohol exposure in adolescent rats	Amygdala	Decreased Kdm6b, increased H3K27me3 at Arc SARE site; Decreased Arc expression; Kdm6b siRNA in CeA causes anxiety and AIE phenotype [193].
Intermittent alcohol vapor exposure in rats	mPFC	Increased KDM6B protein and decreased H3K27me3 [182].
Gestational Day (GD) 7	GD 17 cortex	Increased H3K9me2, and decreased H3K27me3; Altered G9a, Setdb1, Kdm1a, Kdm4c, Uhrf1 and Ezh2 mRNA levels [195].
GD 7–21	Embryonic days (ED) 7.0–14.5	Decreased H3K4me2, H3K4me3; Decreased mRNA levels of Set7/9; Increased G9a mRNA and H3K9me2 [196].
	PD 60–80 from F1-F3 generation ED14.5–PD 7	Decreased H3K4me2, H3K4me3, and Set7/9 mRNA; Increased G9a and setdb1 mRNA, H3K9me2 [197]
PD7 mice	PD7 HP and NC	Increased G9a [198]; Increased degradation of H3K9me2 and H3K27me2 by activated caspase 3 [34] Inhibition of G9a before ethanol treatment rescued degradation of H3K9me2 and LTP and spatial memory deficits [199] Enhanced H3K9me2 and G9a at Arc [200] and Rac1 [201] gene promoters; Reduced H3K9me2 and G9a at Cnr1 gene exon-1 [202]
GD 0–8 days mice	PD87 HP	Increased H3K4me3 at Slcl7a6 gene; Increased Slc17a6 gene and reduced VGLUT2 protein levels [203]
*Macaca nemestrina* (GD 33–46)	DG Ependyma	Increased H3K4me3 at Slcl7a6 gene; Increased Slc17a6 gene and reduced VGLUT2 protein levels [203].
Human prenatal ethanol	Fetal brain ependyma cells	Reduced H3K4me3 [204]
PD7	PD7 7 h after ethanol exposure (NC and Cerebellum)	PD7 7 h after ethanol exposure (NC and Cerebellum) [205].

PD—Postnatal day; GD—Gestational day; ED—Embryonic day; PFC—Prefrontal cortex; HP—Hippocampus; NC—Neocortex; ACC—Anterior cingulate cortex; CCx—cerebral cortex; mPFC—Medial prefrontal cortex; DG—Dentate gyrus; H3K4—Histone3 lysine 4; H3K36—Histone3 lysine 36; H3K9me2—Histone3 lysine 9 dimethylation; H3K27me2—Histone 3 lysine 27 dimethylation; H3K4me2—Histone3 lysine4 dimethylation; H3K4me3—Histone3 lysine4 trimethylation; Set7/9—Set domain histone lysine methyltransferases; Setdb1—Set domain bifurcated1; Slcl7a6—Solute Carrier Family 17 (Vesicular Glutamate Transporter), Member 6. *GIPC1*—PDZ Domain Containing Family Member 1; *BCL2L1*—Bcl-2-like protein 1; UBE1—Ubiquitin Activating Enzyme1; Ezh2—Enhancer Of Zeste 2 Polycomb Repressive Complex 2 Subunit.; BDNF—Brain Derived Neurotrophic Factor; Arc—Activity Regulated Cytoskeleton Associated Protein; AUD—Alcohol use disorder; KDM6b—Lysine Demethylase 6B; H3K27me3—Histone 3 lysine 27 trimethylation; Mt1—Metallothionein 1; Mt2—Metallothionein 2; Setdb1a—Set domain Containing 1a, Histone Lysine Methyltransferase; Setd1b—Set domain Containing 1b, Histone Lysine Methyltransferase; Setdb2—SET Domain Bifurcated Histone Lysine Methyltransferase 2; Setd4—SET domain-containing Protein 4; Setd3—SET Domain Containing 3, Actin Histidine Methyltransferase; Setd6—SET Domain Containing 6, Lysine Methyltransferase; Setd7—SET Domain Containing 7, Lysine Methyltransferase; Set9—SET Domain Containing 9, lysine methyltransferase; Suv39 h1—Suppressor Of Variegation 3–9 Homolog 1; Setdb1—SET Domain Bifurcated Histone Lysine Methyltransferase 1 Prmt6, Protein Arginine Methyltransferase 6; G9a—Euchromatic histone-lysine N-methyltransferase 2; NR2B—N-methyl D-aspartate receptor subtype 2B; cFos—Fos Proto-Oncogene, AP-1 Transcription Factor Subunit/Fos family of nuclear oncogene; Cdk5—*Cyclin Dependent Kinase 5*; FosB—FosB Proto-Oncogene, AP-1 Transcription Factor Subunit/Fos family of nuclear oncogene; LSD1—*lysine-specific demethylase 1; LSD1+8A*—Neuronal-specific isoform, catalyzes the demethylation of the repressive mark H4 K20me2; *CeA*—Central nucleus of the amygdala; MeA—Medial amygdala; PRDM2—PR/SET Domain 2/PR Domain Containing 2, With ZNF Domain; dmPFC—dorsomedial prefrontal cortex; shRNA—short hairpin ribonucleic acid; H3K39Me1—Histone3 lysine 39 monomethylation; H3K39Me2—Histone3 lysine 39 dimethylation; H3K39Me3—Histone3 lysine 39 trimethylation; mRNA—Messenger Ribonucleic acid; Smyd3—SET And MYND Domain-Containing Protein 3; H2AZ—H2A.Z Variant Histone; H2AZK8me1—H2A.Z Variant Histone lysine 8 monomethylation; Ash1l—Absent Small And Homeotic Disks Protein 1 Homolog; Arc SARE—Activity Regulated Cytoskeleton Associated Protein Synaptic Activity Responsive Element; siRNA—Small interfering RNA; AIE—Adolescent intermittent ethanol; Kdmla—Lysine Demethylase 1A; Kdm4c—Lysine Demethylase 4C; Uhrf1—ubiquitin-like protein, containing PHD and RING finger domains 1; VGLUT2—vesicular glutamate transporter 2.

## Data Availability

Not applicable.

## Abbreviations

AD	Alzheimer’s disease
PD	Parkinson’s disease
HD	Huntington’s disease
ALS	Amyotrophic lateral sclerosis
ND	Neurodegenerative
miRNAs	micro RNAs
K	Lysine
HMT	Histone methyltransferases
HMD	Histone demethylases
H3K4me3	Trimethylated Histone3 Lysine 4
MLL2	H3K4-specific histone methyltransferase
H3K9me2	Dimethylated histone H3 lysine 9
GLP/G9a	Histone 3 Lysine9-specific histone methyltransferase
H2B K108	Histone 2B lysine 108
H4	Histone 4
FAD	Fetal alcohol spectrum disorder
Htt	Huntingtin
ESET	ERG-associated protein with SET domain
SETDB1	Histone-lysine N-methyltransferase domain bifurcated 1
CREB	Cyclic AMP response element-binding
CBP	Cyclic AMP response element-binding protein
Ac	Acetylcholine
CHRM1	Cholinergic Receptor Muscarinic 1
PRMT5	Protein arginine methyltransferase 5
H2A	Histone 2A
H4	Histone 4
H4R3Me2	Dimethylated histone 4 arginine3
JMJD6	H4R3Me2 demethylase, PRC2, Polycomb group protein
H3K27Me3	Trimethylated Histone 3 lysine 2
*Hox*	homeobox
Ezh2	Enhancer of Zeste 2
SUZ12	Polycomb repressive complex 2 subunit
Utx	H3K27me3 demethylases
PFC	Prefrontal cortex
CAG	Cystine–Alanine–Guanine
REST/NRSF	RE-1 silencing transcription factor/neuron-restrictive silencer factor
REST	RE-1 silencing transcription factor
BDNF	Brain derived neurotropic factor
JARID1C	Jumonji AT-rich interactive domain 1C
REM	Rapid eye movement
H3K27	Histone 3 Lysine 27
FUS	fused in sarcoma
HDAC	Histone deacetylase
SNCA	alpha synuclein gene
α-SYN	alpha Synuclein
NRF2	Nuclear Factor erythroid-2-Related Factor 2
Hmox1	Heme oxygenase decycling 1
Ngf	Nerve growth factor
Vgf	Nerve growth factor inducible
SYN	Synaptophysin
SH-SY5Y	Human Bone Marrow Neuroblast Cell Line
H3K9me1	Mono methylated Histone 3 lysine 9
iPSCs	Induced Pluripotent Stem Cells
NeuN	Neuronal nuclei
FAS	Fetal alcohol syndrome
FASD	Fetal alcohol spectrum disorders
AUD	Alcohol use disorder
HP	Hippocampus
NC	neocortex PDYN, Prodynorphin
PNOC	Pronociceptin
CIE	Chronic intermittent ethanol
NMDA	N-methyl-D-aspartate
NR2B	N-methyl-D-aspartate receptor 2B subunit
Setd1a	SET Domain Containing 1A, Histone Lysine Methyltransferase
Setd1b	SET Domain Containing 1b, Histone Lysine Methyltransferase
Setdb2	SET Domain Containing b2, Histone Lysine Methyltransferase
Suv39 h1	suppressor of variegation 3–9 homolog 1
Setd4	SET Domain Containing 4
Setd6	SET domain containing 6
Setdb1	SET Domain Bifurcated Histone Lysine Methyltransferase 1
Prmt6	Protein Arginine Methyltransferase 6
AIE	adolescent intermittent ethanol
c-FOS	Fos-related antigen 1
Cdk5	Cyclin Dependent Kinase 5
FosB	Proto-Oncogene, AP-1 Transcription Factor Subunit
TDP-43	transactive response DNA binding protein with M_r_ 43 kD (TDP-43)
LSD1	lysine demethylase 1
CeA	Central nucleus of the amygdala
MeA	medial amygdala
PRDM2)	Histone methyltransferase PR domain containing 2, with ZNF domain
Mag	Myelin-associated glycoprotein
mab	Myelin basic protein
*Mobp*	Myelin-associated oligodendrocytic basic protein
*Plp*	Myelin proteolipid protein
PAE	Prenatal alcohol exposure
ARC	β-endorphin protein in the arcuate
PEE	postnatal ethanol exposure
VGLUT2	vesicular glutamate transporter
CNS	central nervous system
Kmt2	lysine methyltransferase 2E
